# How the Effect of Virtual Reality on Cognitive Functioning Is Modulated by Gender Differences

**DOI:** 10.3390/bioengineering11040408

**Published:** 2024-04-21

**Authors:** Stefania Righi, Gioele Gavazzi, Viola Benedetti, Giulia Raineri, Maria Pia Viggiano

**Affiliations:** Department of Neurofarba, University of Florence, Via di San Salvi 12, 50135 Florence, Italy; gioele.gavazzi@unifi.it (G.G.); viola.benedetti@unifi.it (V.B.); giulia.rainerivr@gmail.com (G.R.); mariapia.viggiano@unifi.it (M.P.V.)

**Keywords:** virtual reality, executive functioning, gender differences, divided attention, visuospatial abilities

## Abstract

Virtual reality (VR) can be a promising tool to simulate reality in various settings but the real impact of this technology on the human mental system is still unclear as to how VR might (if at all) interfere with cognitive functioning. Using a computer, we can concentrate, enter a state of flow, and still maintain control over our surrounding world. Differently, VR is a very immersive experience which could be a challenge for our ability to allocate divided attention to the environment to perform executive functioning tasks. This may also have a different impact on women and men since gender differences in both executive functioning and the immersivity experience have been referred to by the literature. The present study aims to investigate cognitive multitasking performance as a function of (1) virtual reality and computer administration and (2) gender differences. To explore this issue, subjects were asked to perform simultaneous tasks (span forward and backward, logical–arithmetic reasoning, and visuospatial reasoning) in virtual reality via a head-mounted display system (HDMS) and on a personal computer (PC). Our results showed in virtual reality an overall impairment of executive functioning but a better performance of women, compared to men, in visuospatial reasoning. These findings are consistent with previous studies showing a detrimental effect of virtual reality on cognitive functioning.

## 1. Introduction

Virtual reality (VR), which is based on emerging wearable digital devices such as head-mounted displays (HMDs), could be a promising tool not only for implementing games but also for real-life simulations, for example, in test driving and/or in cognitive assessment of neuropsychological patients in ecological settings [1]. However, the real impact of emerging technologies on cognitive functioning is still unclear [1]. In fact, several studies [2,3] raise concerns about how these new technological tools might affect cognitive processing. For example, the “mere presence” of devices like mobile phones can interfere with selective attention (even when the user tries to ignore them) producing deficits in task performance, especially for tasks with greater attentional and cognitive demand [4,5,6,7]. In recent years, a growing quantity of research investigated the relationship between the amount of time devoted to the use of different technological media weekly (media multitasking index)—for the most common uses: listening to music, internet navigation, using social media, viewing video content, etc.—and executive functioning (across three subcategories: inhibition, working memory, and attentional shifting). These studies produced mixed results. Most research has found that high technological media use has detrimental effects on working memory [8,9,10,11], digit span [12], and the ability to inhibit a response or filter out irrelevant information [8,13,14,15,16].

Conversely, other studies reported no performance differences between subjects that use technological media occasionally or for a long time in working memory tasks ([17,18,19] (digit span task); [18] (a reading span task); [20] (for backward digit span and Corsi block tasks)). Some of these studies even reported that the high usage of technological media has a positive effect on selective attention [19] and working memory performance [21].

The fundamental question underlying all these studies is whether—and to what extent—the use of technological media can interfere with our cognitive abilities and ultimately modify our brains. In this vein, a brain imaging study [22] showed that the habitual high use of technological media is associated with an attentional deficit that is directly evident in the functioning of the brain’s attention control circuits. Subjects highly engaged in media multitasking activities indeed exhibited an increased activity in the right prefrontal areas which indicates greater difficulty in recruiting cognitive control resources needed for effective attention and executive functioning. 

The evidence that new technologies can impact our minds is more pressing if we consider VR. The possibility that VR can significantly influence cognitive processing is strictly related to its nature. In fact, VR is a technological tool that simulates reality and our mind is a biological system that has an identical goal: to simulate reality to assess opportunities and threats [23]. Specifically, VR is a highly immersive technological tool, designed to make one lose awareness of reality, and this may modify the sensations, emotions, and attitudes of users, producing an interference in cognitive processing. 

Nonetheless, several studies employed VR to simulate various real-life situations, for example, to assess test driving, road safety [1], learning and skill training in education, and so on, but the scientific literature is still in its early stages of investigating the training effect of immersive VR. In fact, this literature shows mixed outcomes about both the reliability of the use of VR and the real impact on performance. For example, experimental scientific research, which has examined the advantages of utilizing immersive 3D VR rather than a 2D computer for learning and skill training in an educational context, produced inconsistent results [24,25] (for a review, see [26]). Some studies showed a better learning performance in students trained with 3D VR with respect to a 2D computer [27,28] whereas other research showed that the VR produced a greater sense of presence in students but less learning [25,29]. Furthermore, an extensive review [26] concluded that the use of VR training provides training transferability that is not significantly different from traditional training methods. Some authors [26,29] argued that the ineffectiveness of VR training may depend on the fact that VR produces extraneous cognitive load through aspects of the immersive environment that distract rather than enhance the VR training. In fact, VR can create an immersive experience that simulates elements of the real world but it is still an artificial and limited representation of reality. VR technology cannot yet replicate all the subtleties and distinctions of the real world. For example, VR experiences may not capture the full range of physical sensations and may not be able to wholly replicate complex real-world scenarios. This could represent a disruptive element during cognitive processing because interacting with a world perceived as artificial, or not completely real, could inherently represent an additional cognitive load for our mind.

To the best of our knowledge, no study has explicitly explored this possibility, namely, if virtual reality can interfere due to its artificial nature [26,29] with cognitive processing abilities. Understanding whether and to what extent immersion in VR can have detrimental effects while performing other cognitive tasks is also a crucial point for the design of sessions of simulation, learning, and rehabilitation in VR.

Hence, the present study aims to explore if cognitive performance in the domains of working memory, executive functions, and visuospatial abilities can be affected by a concurrent visual task (divided attention) performed in 3D VR via a head-mounted display management system (HDMS condition) rather than on a personal computer with stimuli presented on a 2D screen (PC condition). Therefore, this study also implicitly explores divided attention, which is defined as the ability to execute several tasks simultaneously (dual-task paradigm) [1], in two experimental conditions that differ in the degree of immersivity. We hypothesize that the amount of elaboration required by our brain to perceive the virtual world is cognitively higher for the real world and if this is true it will certainly affect other cognitions. In particular, attention and working memory are the functions most dependent on the load of information.

Our experimental task is a dual-task paradigm where subjects were required to concurrently perform two activities by alternately allocating attention [30]. Allocation of attentional resources is an adaptive process and when two tasks have to be executed together and both involve a high level of cognition and attention, there is an interference that affects task execution [30]. Two major theoretical models have been proposed to explain divided attention: central capacity models [31] and multiple resource models [32]. Central capacity models [31] assume that a central processor allocates undifferentiated attentional resources for different tasks based on their relative difficulty. Whereas attentional models of multiple resources [32] hypothesize the existence of modality-specific attentional resources that allow the simultaneous processing of multiple tasks which involve different sensory modalities (e.g., visual and auditory modalities). 

Performance in a dual task would decline only when the two tasks drain attentional resources from the same sensory modality. Given that our study employs auditory tasks (working memory and executive functions) and a visual task (both in VR and via a computer), we could not expect—following multiple resource models of attention [32]—differences in performance between the two conditions. Therefore, VR would not interfere with the executive performance. Differently, according to the central capacity models [31] and the hypothesis that VR drains more attentional resources (due to the increased cognitive load required to interact with an immersive world perceived as artificial) with respect to a computer, we could predict a worse executive performance in the VR condition.

In addition, possible gender differences in cognitive performance were also explored in conjunction with the use of virtual reality. The decision to include gender differences as a study variable has two foundations. First, some studies [33,34] found that women outperformed men in visual and motor dual tasks as well as in a velocity stereovision task [35] and localization and memory tasks [36]. Second, some research evidenced gender differences in VR experiences. For example, females have also been detected as more sensitive to emotional information in simulated environments [37], describing a higher level of sense of embodiment [38], as well as immersivity when 3D images are presented [38]. The relationship between experienced immersivity and performance [24,39,40] suggested that the superior performance of females might be linked to their higher immersivity experience [41]. However, all these studies focused on navigation within VR and to the best of our knowledge no study has investigated gender differences in executive performance using a dual task assessing divided attention with different immersion conditions, that is to say, VR via HDMS and PC administration.

To achieve these goals we administered a dual paradigm to the subjects, which required them to perform tests either on a computer or in VR. Tests assessed the domains of auditory working memory (digit memory span, forward and backward), executive functioning (auditorily presented arithmetic reasoning test), and visuospatial abilities (visual–spatial intelligence test). Concurrently, participants were engaged in visual exploration of places on a 2D computer screen (PC condition) or in 3D VR (VR condition). In the PC condition participants performed the experiment using an Asus laptop model X540S whereas in the HDMS condition participants wore the Oculus Quest device.

## 2. Materials and Methods

### 2.1. Participants 

A total of 100 volunteers (age range 18–39 years) comprising 52 females and 48 males, all of Italian citizenship, were recruited for this study via posters located in university facilities as well as in recreational clubs. All participants were native Italian speakers with normal hearing, normal or corrected-to-normal vision, and no self-reported history of psychiatric or neurological illnesses and no self-reported use of psychotropic substances. All participants provided informed consent before participating in the study.

### 2.2. Experimental Design 

A dual-task paradigm in a between design was used. Participants were randomly divided into two groups: the PC condition (PC) group (n = 50) and the virtual reality condition (VR) group (n = 50). The PC group (24 females and 26 males) performed tasks using a laptop computer (Asus laptop model X540S, ASUSTeK Computer Inc., Taiwan), a tool familiar to all, while the VR group (28 females and 22 males) used the Oculus Quest virtual reality HDMS (https://www.paginainizio.com/test/index.php accessed on 25 April 2023).

### 2.3. Materials

#### 2.3.1. Digit Memory Span—WAIS-IV [40] 

This test assesses the working memory capacity (WM) of participants through the auditory presentation of progressively longer sequences of a fixed number of digits (3 to 9 digits). The test distinguishes between forward and backward sequences and provides insight into auditory short-term memory and working memory, respectively. Stimuli were composed of couples of sequences of a fixed number of digits (starting from 3 digits). The experimenter read a sequence of digits (1 digit a second) and participants were required to recollect correctly what they heard, in the exact order. After a successful trial (when at least one sequence was correctly recalled), the number of digits in the sequence was increased by one. The participant’s span is the longest number of sequential digits in a successful trial. In the forward version, the sequence had to be recollected in the same order of presentation whereas, in the backward version, participants were required to recollect the items in the exact reverse order.

#### 2.3.2. Arithmetic Reasoning Test

This subtest is adapted from the Wechsler Scale (WAIS-IV [42]). Participants were presented with five arithmetic problems audibly, meant to be solved mentally, and their responses were verbalized. The test assesses attention, logical reasoning, and executive processing skills.

#### 2.3.3. Visual–Spatial Intelligence Test

**Figure 1:**
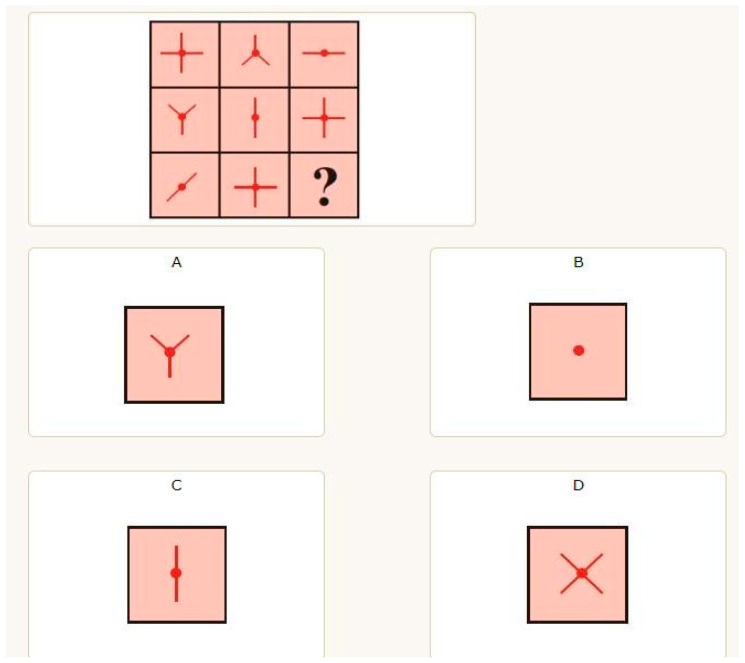
Example of an item of the Visual–Spatial Intelligence Test (https://www.paginainizio.com/test/quiz.php?id=test_intelligenza_visuo_spaziale accessed on 25 April 2023).

A visual–spatial ability test (accessible at: https://www.paginainizio.com/test/quiz.php?id=test_intelligenza_visuo_spaziale accessed on 25 April 2023) was employed in the study to assess the ability to represent, transform, and retrieve symbolic information given by a visual input. Proficiency in memorizing and identifying geometric shapes, along with a broader skill set in appropriately managing information originating from the perceptual space, is essential for this kind of intelligence. We used the first 9 items of the test (which is composed of 18 items). Participants were requested to complete a series of figures which involved the solution of visuospatial problems that engage perceptual–analogical and logical–abstract cognitive processes (see, Figure 1). The participants verbally indicated the letter of the item that completed the pattern.

#### 2.3.4. Immersivity Questionnaire

To tap the subjective experience of immersivity in the PC and VR conditions we adapted a self-report measure of telepresence [43]. This questionnaire was a 9-point Likert scale (Strongly Disagree–Strongly Agree) composed of 8 questions. According to previous research [44], the questions involve diverse metrics for immersivity experience, encompassing (1) subjective assessment; (2) physiometric indicators; (3) virtual-world task performance; (4) natural-world task performance; (5) frame of reference conflict resolution; (6) context reorientation time/degree of disorientation. 

### 2.4. Procedure

Participants were asked to provide three geographical locations (chosen from a list of cities) they would like to visit. Subsequently, participants were placed in front of a laptop in the PC group or wore the Oculus Quest head-mounted display (HMD) device in the HDMS group with the “Google Maps” application open. Then, participants were instructed to search for the first location indicated and they were directed to focus on the screen, keeping their gaze fixed on it, paying attention only to the words provided by the experimenter. Participants were then asked to navigate within the application using the satellite mode, thus virtually visiting the chosen location. Following this, the experimenter administered the digit memory span forward test. Afterwards, participants were invited to search and visit the second place and concurrently the experimenter administered the digit memory span backward test. Participants then explored the third location and simultaneously the arithmetic reasoning test was conducted. Finally, both groups concluded with the visual–spatial intelligence test conducted in the PC or the HDMS condition. Ultimately, all subjects (both PC group and HDMS group) were required to respond to the immersivity questionnaire. All subjects were assessed in a dimly lit room while they sat in a comfortable chair.

### 2.5. Statistical Analysis

For all dependent measures, i.e., digit memory span forward and backward, arithmetic, and visuospatial reasoning accuracy (p), a residual bootstrap [45] ANOVA was run for hypothesis testing with CONDITION (2 levels: PC and HDMS) and SEX (2 levels: M and F) as between-subjects predictors. The same analysis was run also with the “immersivity questionnaire score” as the dependent measure. Significant interactions were further explored by means of a percentile bootstrap comparison (two-tailed) between conditions [46] with Bonferroni adjusted alpha level. In both statistics procedures, 10,000 bootstrap iterations were employed. We choose this statistical technique, specifically the bootstrap method instead of frequentist approaches, as it is more robust. Specifically, bootstrapping does not rely on strict assumptions regarding data distribution as does analysis of variance. It has greater adaptability and robustness, as evidenced by the iterative nature of the analysis [46,47,48,49]. Furthermore, in order to understand whether the immersivity of experience can influence cognitive performance, we performed a bootstrap Pearson correlation between the immersivity questionnaire score and all dependent measures, i.e., digit memory span forward and backward, arithmetic, and visuospatial reasoning accuracy. This analysis was cumulated between conditions. All correlations were two-tailed with Bonferroni adjusted alpha level. Statistical analysis was run using MATLAB (version 2020b; The MathWorks Inc., Natik, Mass, Portola Valley, CA, USA) and R (version 4.3.1; R Core Team (Lucent Technologies, Murray Hill, Australia) 2023) using the “lmboot” [50] and “bootcorci” packages [47]. 

## 3. Results

### ANOVAs

**Figure 2:**
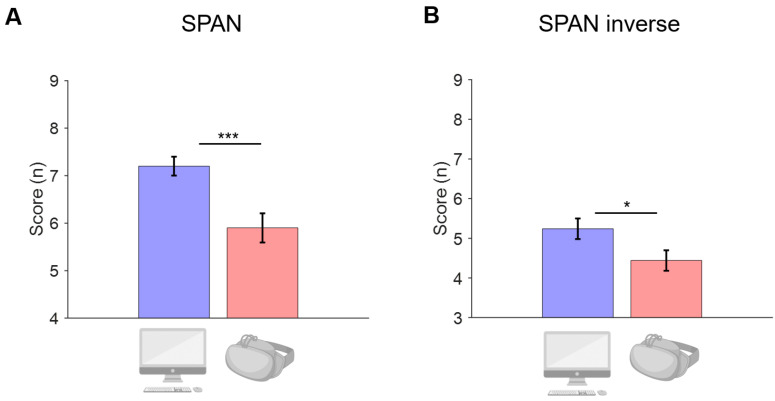
(**A**) Span (forward) and (**B**) span inverse (backward) mean across experiment conditions. Error bars represent ±1 standard error of the mean. *** *p* < 0.001, * *p* < 0.05. Blue bars represent the PC condition and red bars represent the HDMS condition.

A main effect of CONDITION emerged for both digit memory span forward (*p* < 0.001) and backward (*p* = 0.032) measures. In both tasks, the span was longer for the PC condition of the experiment (span: 7.2 ± 1.4; span inverse: 5.2 ± 1.8) compared to the HDMS condition (span: 5.9 ± 2.2; span inverse: 4.4 ± 1.8). Please see Figure 2. 

**Figure 3:**
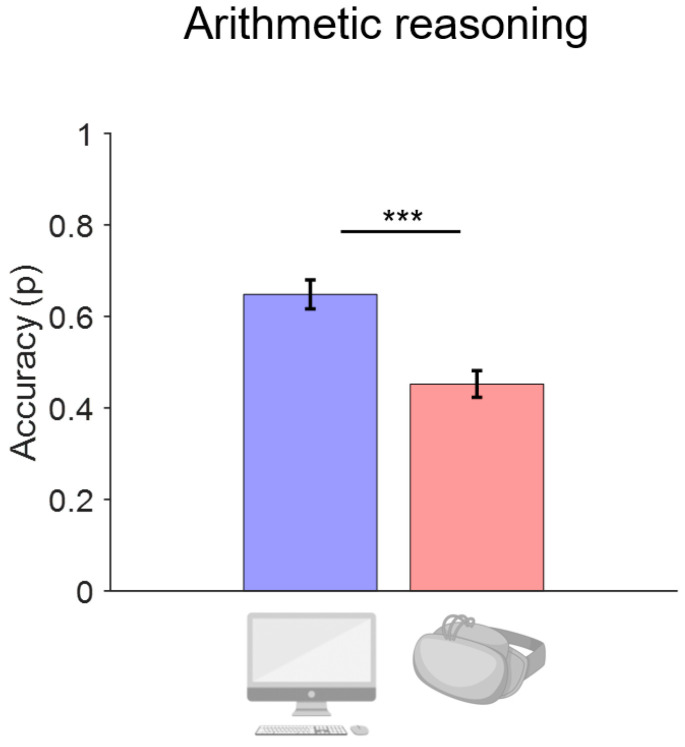
Arithmetic reasoning accuracy (p) results showing mean across experiment conditions. Error bars represent ±1 standard error of the mean. *** *p* < 0.001. Blue bars represent the PC condition and red bars represent the HDMS condition.

A main effect of CONDITION also emerged for the arithmetic reasoning test on the accuracy (*p* < 0.001). The accuracy was higher for the PC condition of the experiment (0.65 ± 0.22) compared to the HDMS (0.45 ± 0.21). See Figure 3. 

**Figure 4:**
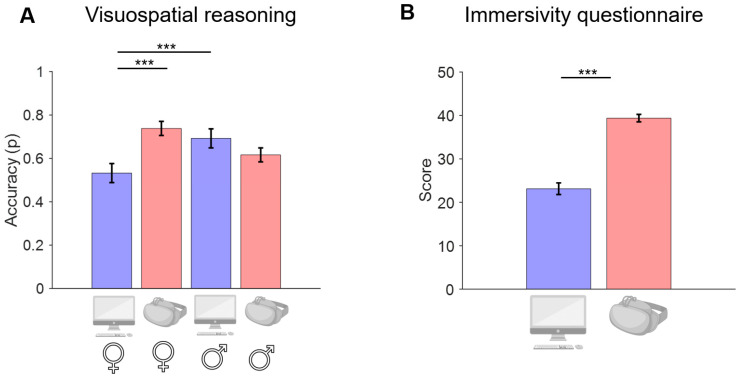
(**A**) Visuospatial reasoning results. Accuracy (p) means across experimental conditions and sex. (**B**) Immersivity questionnaire score across experimental conditions. Error bars represent ±1 standard error of the mean. *** *p* < 0.001. Blue bars represent the PC condition and red bars represent the HDMS condition.

A significant interaction effect of CONDITION * SEX emerged for the visual–spatial intelligence test on the accuracy measure (*p* = 0.001). Post hoc comparisons revealed a significant difference (*p* < 0.001) for females only between the HDMS and PC conditions of the experiment, with a difference estimate (HDMS–PC) of 0.21 (CI = [0.10, 0.31]) indicating higher accuracy in the HDMS condition (0.74 ± 0.17) compared to the PC (0.53 ± 0.22). Moreover, we found a significant difference (*p* = 0.005) between males and females while performing the task on the PC, with a difference estimate (M–F) of 0.16 (CI = [0.05, 0.27]) indicating higher performance for males (0.69 ± 0.18)—Figure 4A.

To summarize, in the PC condition, males outperform females in visuospatial reasoning. At the same time, this difference is compensated in the HDMS condition (we did not find a significant difference here), seemingly due to a greater performance for females in this latter condition.

Finally, a main effect of CONDITION emerged for the immersivity questionnaire scores (*p* < 0.001) with the PC being perceived as less immersive (23.1 ± 9.4) compared to the HDMS (39.4 ± 6.1) experience—Figure 4B.

Finally, the correlation between the immersivity questionnaire and the span score was significant (estimate = −0.30, C.I. = [−0.47; −0.13], *p* < 0.001).

## 4. Discussion

Our primary aim was to investigate whether and to what extent the interaction with VR (head-mounted display (HMD) device), compared to the use of a device like a computer (PC condition), can interfere with executive functioning in a dual task.

The results confirmed the hypothesis of a detrimental effect of VR on concurrent cognitive performance and also provided interesting insights into gender differences. In fact, we found that in the HDMS condition all subjects performed worse, with respect to the PC condition, in tasks that involve executive functions such as digit memory span forward and backward performance and arithmetic reasoning.

Additionally, we found in the digit memory span forward test a better performance in men, with respect to women, consistent with previous literature [51]. However, this advantage is not retained in the HDMS condition, where both women and men showed a decrease in performance. Similarly, the VR condition also interfered with the digit memory span backward test performance and with the arithmetic reasoning test, indicating that not only auditory working memory but also central executive information processing abilities are compromised in the VR condition.

Since these cognitive tasks were performed concurrently with a visual navigation task on technological devices (computer or HMD device) our study also explores divided attention. Our results showed that 3D VR interferes more than a 2D computer with the ability to distribute the attentional resources between two tasks involving different sensory modalities (visual exploring and auditorily presented working memory tasks). This evidence agrees with the central capacity model [31] which hypothesizes that a central processor assigns homogeneous attentional resources for different tasks on the basis of both their cognitive loads and their automatic or controlled attentional characteristics [52]. Since the central capacity theory explicitly states that the cognitive load is a crucial factor in allocating greater or lesser attentional resources, the evidence of a greater impairment in test performance while subjects were immersed in VR, compared to the PC condition, could be an indirect indication that VR induces cognitive overload in participants. This could have a dual explanation. On one hand, the 3D world that is artificially recreated in VR does not fully correspond to the complexity of the real world, and this can overload the cognitive system that perceives itself as interacting with an artificial creation [26,29]. On the other hand, VR, being a highly immersive world, may cause users to lose the ability to interact with external stimuli effectively, resulting in a decrease in cognitive performance. This interpretation is also consistent with previous research which revealed that the presence of competing external distractors during the initial working memory encoding period (0–500 ms) greatly reduces working memory performance [53,54]. In this vein, the highly immersive VR experience would produce a greater interference because it competes with targets for limited representational space in working memory [53,55,56,57] as confirmed by the negative correlation we found between the immersivity questionnaire and the span score. Consistent with this hypothesis, our participants rated the VR experience as more immersive than the PC experience.

To the best of our knowledge, this is the first study that explicitly investigated whether and to what extent the interaction with 3D VR, compared to the use of a 2D device like a computer (PC condition), can interfere with executive functioning in a dual task, therefore it is not possible to compare our results with previous literature. The most coherent literature that one can attempt to draw parallels with is that which has investigated the effects of VR on learning training in educational contexts. With the above-mentioned studies in mind, our findings of impaired executive performance in VR are consistent with the studies that have found that educational training with VR may have negative effects on knowledge and transfer [25,58,59] compared to less immersive media.

The second purpose of this work was to shed light on gender differences in cognitive performance in a dual task concurrent with the use of VR. Unlike previous studies [33,34] (Stoet, et al., 2013; Ren, et al., 2009) which found a superiority of women compared to men in dual visual and motor tasks [35,36], we did not find performance advantages for women in the ability to divide attention.

However, remarkably, our results evidenced that the performance in the visual–spatial intelligence test is modulated by the interplay between technological conditions (PC and HDMS) and gender, with men outperforming women only for the PC condition. At the same time, women showed a worse performance in the PC compared to HDMS condition, while men did not show differences. Notably, this task differed from the others because it no longer involved divided attention but rather a total immersion in the online task on the device. For the PC condition, a web page was simply opened with the visual–spatial intelligence test in which participants were asked to view a sequence of images and indicate the correct one out of four alternatives. Within the HDMS condition, the procedure was the same, but the web page was displayed larger in front of the participant through the HMDS as if it were a luminous board that could be interacted with. The hypothesis in this case was that performance in 3D VR would be better than in the 2D PC condition because participants would be in a state of complete immersion in the task without concurrent activities. This hypothesis was confirmed only in women, while men’s performance was better with respect to women in the PC condition, consistent with literature that often emphasizes greater visuospatial abilities in men compared to women [60,61,62]. A meta-analysis conducted on 286 data samples highlights consistent and stable gender differences in three-dimensional object mental transformation. In some spatial ability tasks, women have advantages, while men perform better in mental rotation, spatial perception, and spatial visualization [63]. What is truly interesting, however, is what happens in the HDMS condition, in which women’s performance increases in accuracy compared to the PC condition. Regardless of performance, several behavioral and neuroimaging studies have highlighted that women tend to rely more than men on working memory and cognitive control processes to perform visuospatial tasks [64,65,66,67,68]. This evidence suggests a compelling intersection between cognitive processes and gender differences. Visuospatial abilities, crucial for tasks such as navigation, object recognition, and mental rotation, involve capacities such as perceiving, analyzing, and manipulating visual information in space. When considering a scenario represented in VR, as opposed to a traditional computer monitor (PC condition), an environmental factor is introduced that may influence cognitive strategies, fostering the utilization of women’s strategies in evaluating spatial information. Virtual reality, with its more immersive and dynamic environment, may demand different cognitive processes compared to traditional computer-based assessments. For this reason, we hypothesize that the observed differences in results may reflect not only disparities in cognitive abilities but also variations in cognitive styles employed to assess distinct scenarios. In other terms, women appear to exhibit greater flexibility in interacting with different types of spatial information, whereas men may consistently approach tasks in a similar manner, evaluating items in a virtual setting as they would do in a traditional setting.

It is crucial to acknowledge the significance of individual differences within genders, as generalizations may not fully capture the spectrum of cognitive diversity. Additionally, the observed gender differences could be influenced by a combination of biological, cultural, and environmental factors that were not totally controlled in the present study. Further research is warranted to explore and elucidate these potential contributing factors.

Given that this study demonstrates that VR can interfere with cognitive task performance by influencing the ability to allocate divided attention—likely due to cognitive overload associated with the highly immersive yet perceived as artificial nature of VR—a result of our work is the suggestion to implement VR scenarios that are increasingly similar and adherent to the subtle differences of the real world.

To effectively address this issue, one compelling strategy may involve harnessing the capabilities of deep learning techniques. By capitalizing on the distinctions observed in divided attention within the confines of our present study, there lies the potential to fine-tune virtual stimulation protocols to mitigate these effects effectively. Such an approach draws parallels to the insights gleaned from research in deep-fake recognition algorithms and human–robot and human–environment interactions [69,70,71] (Badia et al., 2022; Heidari et al., 2023; Heidari et al., 2024). Consequently, it is imperative that forthcoming investigations delve deeper into this promising avenue, probing its applicability across a broader spectrum of cognitive processes. By doing so, researchers can not only refine our understanding of how deep learning can shape virtual experiences but also unlock novel strategies for optimizing human–computer interaction in diverse contexts.

## Data Availability

Data are available from the corresponding author upon request.
